# Food Adulterations

**Published:** 1887-05

**Authors:** 


### FOOD ADULTERATIONS.

### THE PUBLIC HAS POWER IN ITS HANDS TO EFFECT REFORM.

The chemists of the Department of Agriculture, under direction of the Commissioner, have, for a year or more, been investigating the matter of food adulteration in this country, and a report is in process of preparation. That portion relating to spices and condiments prepared by Mr. Clifford Richardson is already completed. Mr. Richardson's investigations show that no other kinds of human food are adulterated to such an extent. Of twenty samples of ground cloves only two were pure. The others had suffered the extraction of their essential oils, and had been polluted by the addition of clove stems, allspice, and husks of various kinds. Of eight samples of Cayenne pepper only one was pure.

Of ten samples of mustard, none were pure unchanged mustard, though several had only suftered the loss of their fixed oil ; the others contained quantities of wheat flour, the spurious matter being in some cases two thirds of the compound. This made it necessary to add tumeric acid (harmless) to restore the mustard color. Ten samples of allspice were examined, eight of which were pure. Four samples of cassia were all pure. Of ten samples of ginger four were pure. Only one out of thirteen samples of black pepper was found to be what it purported to be. A specimen sent from Baltimore to a man who had an army contract was almost entirely spurious. Cayenne pepper, black pepper husks, and mustard hulls were used to give flavor and pungency, while "body" was supplied by ground beans and rice, and color by charcoal. Two samples of white pepper out of five were pure ; two samples of mace out of five were pure, and of three samples of nutmeg examined all were pure.

Mr. Richardson's experience leads to the conclusion that the public has the power in its own hands to effect a reform in the matter of spice adulteration. The quality of the article is usually fixed by the retailer, who names the price he is willing to pay. The grinder thereupon estimates the amount of pure spice he can afford to put in, and fills out the order with refuse. Mr. Richardson incidentally mentions a New York spice grinder who, within a short time worked off 5,000 pounds of cocoanut husks. The ground article, which sells for less than the pure and unground, needs no test to prove it spurious. Usually when he demanded a pure article and called upon a first class grocer for it he got it.

Considerable space is given in the report to the operation of laws at home and abroad relating to the adulteration of food, while the methods of detection, both popular and scientific, are treated exhaustively.

